# Anterior arthroscopic-assisted fixation of posterior cruciate ligament avulsion fractures

**DOI:** 10.1186/s40001-015-0177-6

**Published:** 2015-10-29

**Authors:** Wei Huang, Xuan Gong, Mishra Rahul, Shukla Priyanka, Changdong Wang, Xi Liang, Guoliang Ding, Ning Hu

**Affiliations:** Department of Orthopaedic Surgery, The First Affiliated Hospital of Chongqing Medical University, Chongqing, 400016 China; Department of Outpatient, Chongqing Zhongshan Hospital, Chongqing, 400013 China; Department of Biochemistry and Molecular Biology, Molecular Medicine and Cancer Research Center, Chongqing Medical University, Chongqing, 400016 China; Department of Orthopaedic Surgery, The Second Affiliated Hospital of Baotou Medical College of Inner Mongolia University of Science and Technology, Baotou, 014030 China

**Keywords:** Posterior cruciate ligament, Avulsion fractures, Arthroscopy, Fixation

## Abstract

**Background:**

Avulsion fractures of the tibial insertion of the posterior cruciate ligament (PCL) have always been regarded as rare injuries. The tibial attachment of the PCL is located in an area, which is difficult to access.

**Hypothesis:**

To verify the effects of anterior arthroscopic fixation of PCL avulsion fractures.

**Methods:**

18 patients with PCL avulsion fracture were included. The inclusion criteria were: (1) the fracture fragment size was greater than 20 mm; (2) surgery in the acute phase of fractures (<3 weeks). The intervention was anterior arthroscopic fixation of fractures. Outcome variables included posterior laxity assessment with KT2000 arthrometer, posterior sag sign, the quadriceps activation test, the reverse Lachman, posterior stress X-rays, range of motion, and the IKDC form assessment.

**Results:**

Complete osseous union showed in all cases during the follow-up (24–49 months). The posterior laxity assessment demonstrated slight posterior tibia translation (<5 mm) on the femur in 1 patient at 89 N and in 2 at maximal testing. All were stable on the posterior sag sign, the quadriceps activation test, the reverse Lachman and posterior stress X-rays. Two had loss of flexion of about 10° (grade B). Others showed a full range of knee motion. According to the IKDC form assessment, 16 patients were classified as grade A and 2 were classified as grade B. 16 of 18 patients were absolutely pain free, and there was general satisfaction on pain questionnaire. All the patients returned to their pre-injury knee function. No revision surgery was performed.

**Conclusions:**

The anterior arthroscopic-assisted fixation guided with a tibial PCL guide is a simple and feasible alternative for treating PCL avulsion fractures when the fragment size is larger than 20 mm.

**Level of evidence:**

Case–control study, Level III.

## Background

Avulsion fractures of the tibial insertion of the posterior cruciate ligament (PCL) have always been regarded as rare injuries [1–3]. The PCL serves as the primary restraint against posterior tibial displacement and together with the anterior cruciate ligament regulates external rotation of the knee during extension [4, 5]. PCL avulsion fractures are most commonly the result of dashboard injury, sudden hyperextension in conjunction with associated valgus or varus forces [6–9]. Surgical treatment of displaced PCL avulsion fractures is essential to prevent non-union or malunion, which can cause knee instability, and also surgical repair is done to prevent OA and knee pain [10]. The tibial attachment of the posterior cruciate ligament is located in an area which is difficult to access [11, 12]. Several techniques of open reduction and internal fixation under direct vision from a posterior approach are acceptable options [8, 10]. The purpose of our study is to verify the effects of anterior arthroscopic fixation with one or two cannulated, partially threaded screws for PCL avulsion fractures, in circumstances where the acute avulsion fragment is large.

## Methods

### Patients

All study procedures were approved by the hospital ethics committee. The study only recruited patients who were mentally competent and gave their consent to participation pre-operatively. The study was carried out from Jan 2010 to Nov 2012. The main inclusion criteria were: (1) fragment size of the PCL avulsion fractures of the tibia greater than 20 mm, as Kim et al. recommended [11]; (2) surgical reduction and fixation was done in the acute phase of fractures (<3 weeks). Patients with serious injury associated with other ligament and supporting structures (likely to affect the final outcome) or an occult midsubstance injury of the avulsed PCL were excluded from the study group (Table 1).

**Table 1:** Inclusion and exclusion criteria

No	Inclusion criteria	Exclusion criteria
1	Fragment size of the PCL avulsion fractures of the tibia greater than 20 mm	Patients with serious injury associated with other ligament and supporting structures
2	Surgical reduction and fixation was done in the acute phase of fractures (<3 weeks)	An occult midsubstance rupture of the avulsed PCL

The diagnosis in all cases was based on history, physical examination and imaging studies. A standard preoperative assessment of each case was composed of a clinical examination to define the instability and other associated problems. Routine radiography of the knees showed the fracture. CT scan was also performed to further delineate the bony injury. All the patients were also examined with MRI and arthroscopy to rule out an occult midsubstance injury of the avulsed PCL, ACL injury or injury of the posterior lateral corner. Pre and postoperative posterior instability was determined clinically by performing the posterior drawer test and radiographically by performing posterior drawer stress radiographs. Measurement of the posterior translation with the KT-2000 arthrometer (MEDmetric, San Diego, California) was performed as described by Daniel [13].

### Surgical technique

Each patient was positioned supine with the lower extremity held in 30° flexion. A pneumatic tourniquet was applied. Standard knee arthroscopy was performed with anterolateral and anteromedial portals using a 30° 4.0 mm arthroscope. An additional posteromedial or posterolateral portal was used for a wider and better view. Hematoma, fibrin clots, fat pad and soft tissue interposed in the fracture bed were removed with a motorized shaver that was passed through the above-mentioned portals. Careful probing was then made to identify other associated problems. If feasible, associated meniscal lesions were primarily accessed with suture; if not, the meniscal tear was partially resected. The reduction and temporary fixation of the avulsion fragment could be evaluated using a probe that was passed through the posteromedial or posterolateral portal.

**Fig. 1:**
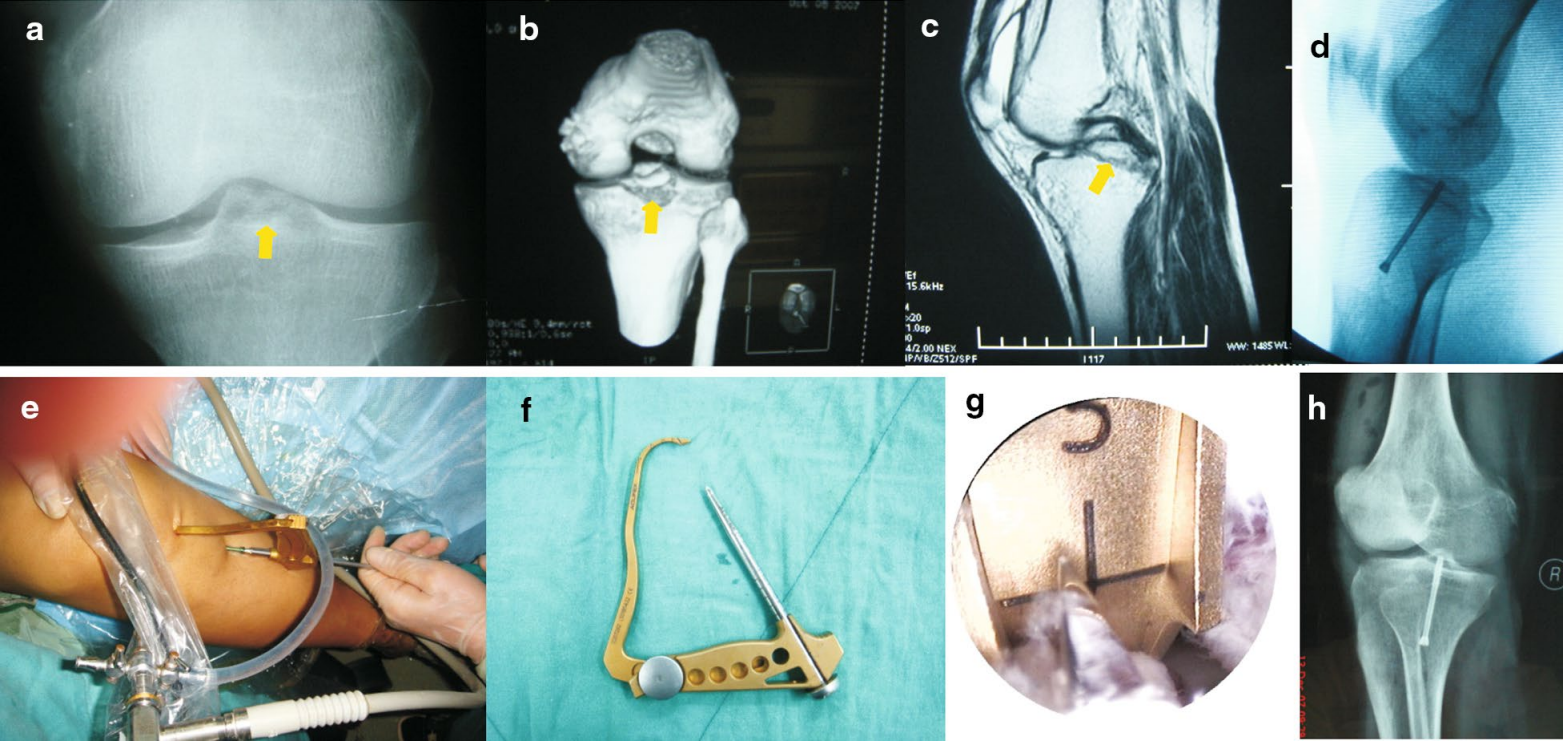
PCL avulsion fracture of a 26-year-old male was fixed anteriorly under arthroscopy with one partially threaded cannulated screws. **a**, **b** X-ray and CT showed the tibial intercondylar eminence fracture. **c** MRI showed the tibial intercondylar eminence fracture of PCL insertion. **e**–**g** The tibial PCL guide (Acufex) was used to secure the fracture fragment and guide one kirschner wire. **d**, **h** The position of one partially threaded cannulated screw was confirmed under image intensifier and X-ray examination after operation. *Arrow* pointed the fracture

**Fig. 2:**
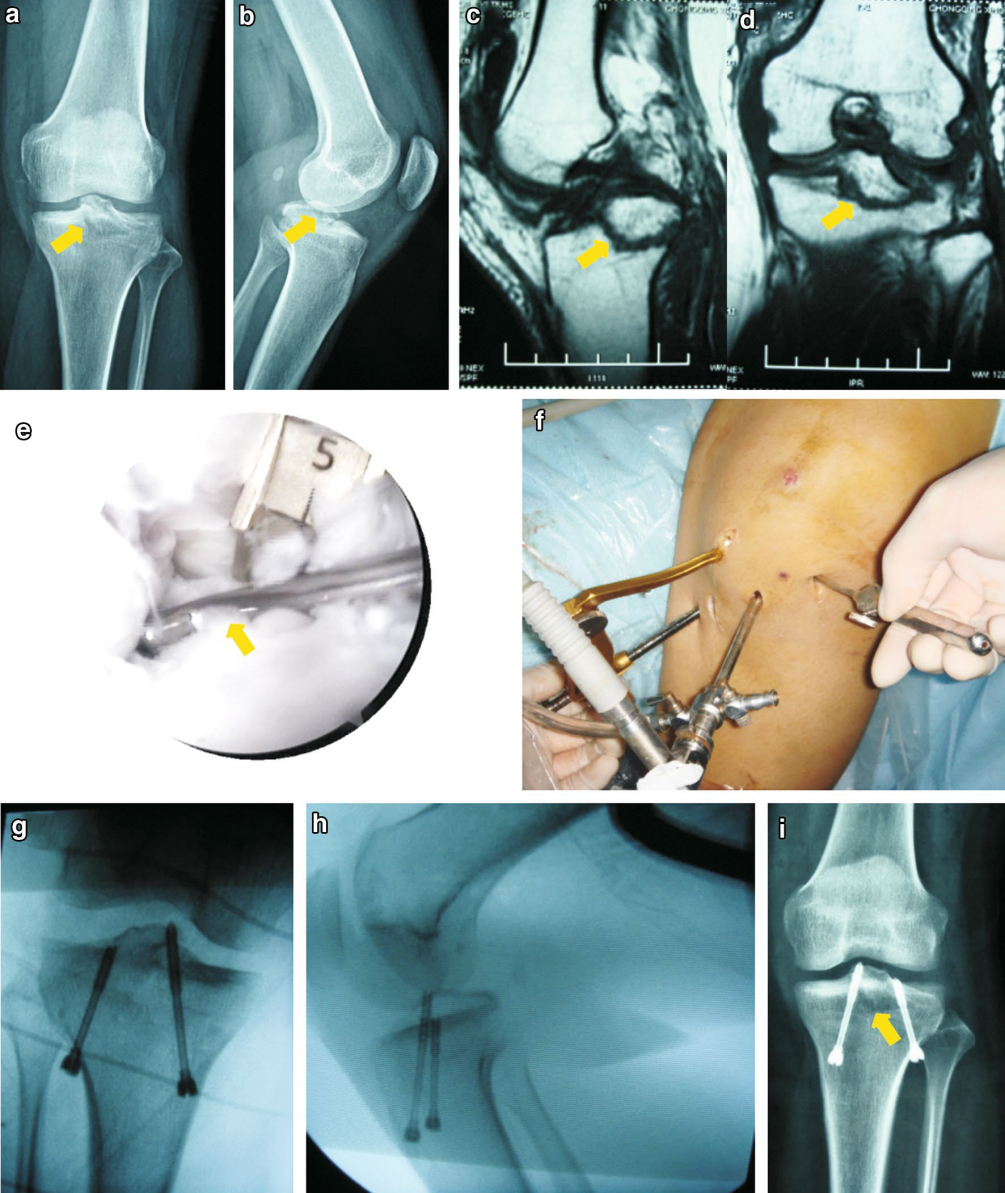
PCL avulsion fracture of a 32-year-old female was fixed anteriorly under arthroscopy with two partially threaded cannulated screws. **a**, **b** X-ray and CT showed the tibial intercondylar eminence fracture. **c**, **d** MRI confirmed the fragment size and displacement of the PCL avulsion fracture. Fragment involved the medial and lateral tibial plates. **e**, **f** Standard knee arthroscopy was performed with anterolateral and anteromedial portals using a 30° 4.0 mm arthroscope. The tibial PCL guide (acufex) and the probe were used to secure the fracture fragment and guide kirschner wires. **g**–**i** The position of two partially threaded cannulated screws was confirmed under image intensifier and X-ray examination after operation. *Arrow* pointed the fracture

The tibial PCL guide (Acufex) was used to further secure reduction of the fracture fragment and guide one or two 2.0-mm Kirschner wires for 4.5-mm cannulated partially threaded screws. It was helpful to perform this in anterior drawer position. Kirschner wires were drilled through the guide from the anterior tibial cortex into the lateral edge of the PCL avulsion site for temporary fixation. Exact positioning of the tip of the K-wire was checked fluoroscopically. Once position of the wires and reduction was confirmed under an image intensifier, a bone tunnel was then made and an appropriately sized partially threaded cannulated screw was used. Depending on fragment size, one or two screws were chosen. These screws were inserted to lag and stabilize the bony fragment. Satisfactory fixation and compression of the avulsed fragment were ensured. Radiographic evaluation demonstrated proper position of the screws with all screw threads within the avulsed bony fragment. Finally, satisfactory fixation of the avulsed fragment should be determined radiographically. Tension of the PCL should be examined with palpation immediately to ensure the perfect fixation (Figs. 1, 2).

### Postoperative and follow-up protocol and measurements

Postoperatively, a controlled hinge knee brace was worn to control motion for 6 weeks and gradually increase range of knee motion [14]. We typically performed only prone passive range of motion for these patients to protect the PCL. Partial or full weight-bearing was not permitted during the first 6 weeks. Mobilization was guided by a physiotherapist. While wearing the brace, patients were encouraged to start patellar mobilizations, quadriceps isometric strengthening exercises, and range of motion exercises, as well as walk with the aid of crutches. Patients were assessed clinically and radiographically at 6 weeks, 3 months, 6 months, 1 year and 2 years later.

The International Knee Documentation Committee (IKDC) objective form was used to evaluate results such as swelling, range of knee movement, jerk test, Lachman test, and sagittal and frontal plane laxity compared to the normal contralateral knee [15]. The posterior laxity was measured with a KT-2000 arthrometer at lease 6 weeks after operation. Other PCL tests included the posterior sag sign, the quadriceps activation test, and the reverse Lachman. Radiologic examination included anteroposterior and lateral X-rays of the knee for evaluation of the fracture union. And radiographic stress test can ensure the final stability after bone healing. This objective described the instability postoperatively and implants were removed easily once bone healing was achieved.

## Results

During the study, a total of 18 patients (13 males and 5 females) were included. The median age of the patients was 28 years (range 20–42 years). The injury was caused by sport activities in 6 patients and by motorcycle accidents in 12 patients. The sport activities resulted in a twisting injury to the knee in 2 patients and a hyperflexion injury in 4. 10 of the motorcycle accidents resulted in direct impact to the proximal part of the tibia while the knee was flexed. In the remaining 2 motorcycle accident patients, the mechanism of injury could not be specified because the patients were unconscious when the injury occurred. All patients underwent surgery within 1 week after trauma (average 4.8 days, range 1–7 days). Examination under anesthesia before the surgery revealed grade-II posterior instability in thirteen knees and grade-III in five knees. The average surgery time was 35 min (range 21–55 min). There were no complications such as neurovascular injuries, marginal skin necrosis and superficial or deep infection. All patients were followed up on average for 34 months (range 24–49 months). In all cases, radiographic examination showed complete osseous union without any loss of reduction, at an average of 3 months after the procedure.

The assessment of posterior laxity with the KT2000 arthrometer demonstrated slight posterior tibial translation on the femur (<5 mm) only in 1 patient at 89 N and in 2 patients at maximal testing. All knees were stable on the posterior sag sign, the quadriceps activation test, and the reverse Lachman. Posterior stress X-rays in the follow-up period objectively described the stability. Only two patients had loss of flexion of about 10° (grade B). Remaining patients showed a full range of knee motion, without any extension limitation at the final follow-up. According to the IKDC form assessment, 16 patients were classified as grade A and 2 were classified as grade B. 16 of 18 patients were absolutely pain free, and there was general satisfaction on pain questionnaire. All the patients returned to their pre-injury knee function. Implants were removed easily in all cases, and no revision surgery was performed.

## Discussion

The posterior cruciate ligament plays an important role in knee stabilization [16, 17]. Avulsion fractures constitute a small sub-group of PCL injuries [2]. Although more and more reports have been published about PCL avulsion fractures and their treatment, there is still no consensus on how to treat this type of injury [18].

Most authors suggested surgical fixation [2, 19], although some surgeons recommend conservative treatment [20]. Surgical treatment of displaced PCL avulsion fractures is essential to prevent non-union or malunion, which can cause knee instability [21]. Previous approaches commonly recommend division of the medial head of gastrocnemius across its muscle fibers, first described by Trickey in 1968 [22], which enhances exposure of the PCL avulsion, but inevitably leads to postoperative weakness of the muscle [16]. Burks and Schaffer’s approach uses the interval between the medial gastrocnemius and the semimembranosus [23]. This approach never involves division of the medial head of gastrocnemius; however, the posterior capsule and the avulsed bony fragment are not well visualized. Also it is difficult to place a screw perpendicular to the fracture plane [16].

Today, the trend has shifted towards arthroscopically assisted minimally invasive techniques [8, 24, 25]. Martinez-Moreno et al. first advocated the possibility of arthroscopic treatment for PCL avulsion fractures; they performed an experimental percutaneous fixation technique with the use of arthroscopic visualization to fix PCL avulsion fractures in cadaveric knees [26]. Kim et al. demonstrated that PCL avulsion fractures can be fixed with the use of arthroscopic methods; they used a variety of methods for fixation including wires, loop sutures and screws. The choice of fixation method was based on the size of the avulsed fragment. Small bone fragments (<10 mm) with comminution were fixed with the use of multiple sutures. Medium-sized fragments (10–20 mm) were fixed with Kirschner wires. Large single fragments of bone (>20 mm) that involved the condyles were fixed with one or two cannulated screws [11]. Fixation with sutures, K-wires, staple, or screws was good, but each technique has its own disadvantage [27]. Major problems include loss of reduction, residual anterior laxity, loss of extension, and non-union [3, 28, 29]. Depending on the fragment size, screws are chosen. First, the tibial PCL guide (Acufex) was used to lag and compress the fragment. Then, one or more screws were inserted to stabilize the fragment accordingly. The function of the screws is to stabilize the bony fracture fragment. It is not necessary that all screw threads should be within the avulsion fragment. Our aim was to achieve stabilization of avulsed fragments.

Our surgical technique has several advantages: (1) it is a simple, valid arthroscopic technique where direct visualization is feasible. (2) It allows haemarthrosis wash, fracture inspection, and removal of interposed tissue. (3) It only needs a PCL reconstruction tibial guide and probe to reduce the fragment and subsequent drill pins to fix the fragment temporarily. (4) Cannulated partially threaded screws are chosen, which allow them to be precisely located through the guide pin and thus achieve a stable fixation with compression between the bony fragment and the proximal tibia. (5) The cannulated screws are noticeably easier to remove. (6) Check exact positioning of the tip of the K-wire fluoroscopically; it can eliminate risk of neurovascular injury. Indications for this technique are as follows: (1) As Kim et al. recommended [11], fragment size should be large (>20 mm), if cannulated screw is chosen. (2) Surgical reduction and fixation in the acute phase is indicated to reduce the fragment conveniently and prevent non-union [5]. The disadvantages are limitation of using for small or comminuted fragments. The limitations of this study include the limited number of patients and the lack of a control group. However, unlike previously reported studies, our study established clinical outcomes of surgery.

## Conclusions

Anterior arthroscopic-assisted fixation guided with a tibial PCL guide is a simple and feasible alternative for treating PCL avulsion fractures when the fragment size is larger than 20 mm.
